# CSPG4: A Target for Selective Delivery of Human Cytolytic Fusion Proteins and TRAIL

**DOI:** 10.3390/biomedicines5030037

**Published:** 2017-06-28

**Authors:** Sandra Jordaan, Shivan Chetty, Neelakshi Mungra, Iris Koopmans, Peter E. van Bommel, Wijnand Helfrich, Stefan Barth

**Affiliations:** 1 South African Research Chair in Cancer Biotechnology, Institute of Infectious Disease and Molecular Medicine (IDM), Department of Integrative Biomedical Sciences, Faculty of Health Sciences, University of Cape Town, Cape Town 7925, South Africa; sandrajordaanibms@gmail.com (S.J.); shivan.chetty@gmail.com (S.C.); mngnee002@myuct.ac.za (N.M.); 2 Department of Surgery, Laboratory for Translational Surgical Oncology, University of Groningen, University Medical Center Groningen, 9713 Groningen, The Netherlands; i.koopmans@umcg.nl (I.K.); p.e.van.bommel@umcg.nl (P.E.v.B.)

**Keywords:** cancer, immunotherapy, CSPG4, HMW MAA, MCSP, antibody drug conjugates, immunotoxins, targeted human cytolytic fusion proteins (hCFP), ETA, MAP tau, angiogenin, TNF ligands, TRAIL

## Abstract

Chondroitin-sulfate proteoglycan 4 (CSPG4) is a transmembrane glycoprotein overexpressed on malignant cells in several cancer types with only limited expression on normal cells. CSPG4 is implicated in several signaling pathways believed to drive cancer progression, particularly proliferation, motility and metastatic spread. Expression may serve as a prognostic marker for survival and risk of relapse in treatment-resistant malignancies including melanoma, triple negative breast cancer, rhabdomyosarcoma and acute lymphoblastic leukemia. This tumor-associated overexpression of CSPG4 points towards a highly promising therapeutic target for antibody-guided cancer therapy. Monoclonal αCSPG4 antibodies have been shown to inhibit cancer progression by blocking ligand access to the CSPG4 extracellular binding sites. Moreover, CSPG4-directed antibody conjugates have been shown to be selectively internalized by CSPG4-expressing cancer cells via endocytosis. CSPG4-directed immunotherapy may be approached in several ways, including: (1) antibody-based fusion proteins for the selective delivery of a pro-apoptotic factors such as tumor necrosis factor-related apoptosis-inducing ligand to agonistic death receptors 4 and 5 on the cell surface; and (2) CSPG4-specific immunotoxins which bind selectively to diseased cells expressing CSPG4, are internalized by them and induce arrest of biosynthesis, closely followed by initiation of apoptotic signaling. Here we review various methods of exploiting tumor-associated CSPG4 expression to improve targeted cancer therapy.

## 1. Introduction

**Figure 1:**
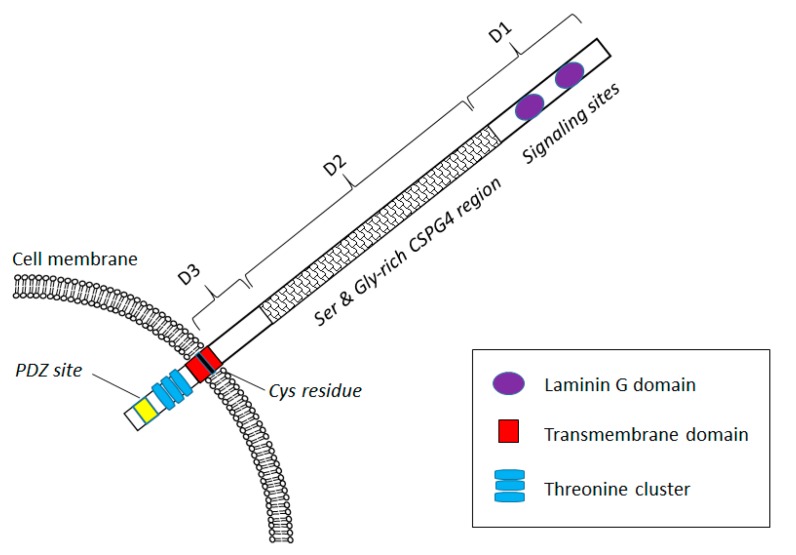
Schematic representation of Chondroitin sulphate proteoglycan 4 (CSPG4) protein domain organization. The CSPG4 protein consists of three extracellular domains (D1 to 3), a transmembrane region (red) and a short cytoplasmic domain. Ligand binding sites for laminin G are indicated in D1 (purple ovals). The extracellular domain is variably subject to glycosylation.

Chondroitin sulphate proteoglycan 4 (CSPG4), alternatively known as melanoma-associated chondroitin-sulphate proteoglycan (MCSP) or high molecular weight melanoma-associated antigen (HMW MAA), is a membrane-integral chondroitin sulphate (CS)-modified glycoprotein-proteoglycan (Figure 1). The amino acid sequence of CSPG4 is highly species conserved, showing over 80% homology with the rat neuroglial-2 proteoglycan (NG2) and the mouse AN2 antigen [1]. The 250 kD glycoprotein core protein is composed of a large CS-modifiable extracellular region, a 45-amino acid hydrophobic transmembrane region and a short 75-amino acid cytoplasmic region [2,3].

The extracellular region of CSPG4 consists of three domains (D1–3), with D1 most distal from the membrane and D3 most proximal to the cell membrane. Each of these domains has distinct structural and functional properties, including binding domains for growth factors and other ligands that constitutively activate mitogenic pathways and transcription [3,4]. The N-terminal domain D1 is characterized by various signaling regions, including a laminin globular (G) domain. D2, the largest extracellular domain, is rich in serine and glycine and includes collagen V and VI binding sites. Characteristically, this domain also comprises several sites for N- and O-linked glycosylation and chondroitin sulfate glycosaminoglycan (CSGAG) modification. CS addition takes place in the Golgi apparatus and the degree of glycosylation can increase the size of the protein to over 500 kD [5]. This modification is seemingly not required for signal transduction functionalities of the CSPG4, although it does mediate differential ligand binding [6]. D3, a small globular region, is the most C-terminal of the extracellular part of CSPG4 and is exclusively subject to N-linked glycosylation.

The cytoplasmic tail includes ERK and PKCα phosphorylation sites (in the form of a threonine cluster region) [7], as well as a PDZ-motif involved in anchoring the membrane-bound CSPG4 to the actin cytoskeleton. Downstream, threonine phosphorylation of this intracellular region of CSPG4 by ERK/PKCα activates cell proliferation and survival pathways (such as the PI-3K/AKT/mTOR pathway), increases cell motility and epithelial-mesenchymal transition (via FAK and Rho family proteins, Rac1 and cdc42), as well as promoting hormone/radiation therapy resistance (via activated cdc42, or ACK1) [5,8,9].

The presence of the above-mentioned extracellular ligand binding domains and intracellular signaling motifs implicates CSPG4 in the regulation (and deregulation) of cell cycle pathways, promoting oncogenic transformations such as epithelial-mesenchymal transition (EMT). Cells that should become terminally differentiated are thus driven by inappropriate expression of CSPG4 to develop characteristics similar to stem cells, such as self-renewal and motility. The relationship between normal CSPG4 expression and tumor-related overexpression will be discussed in the following section. Regulation of CSPG4 expression appears to depend on post-translational modification—DNA hypomethylation of CpG islands in the *CSPG4* promoter has been proposed as potential cause for deregulation of CSPG4 expression, resulting in sustained overexpression in cells that normally would have downregulated CSPG4 levels [10].

### CSPG4 Expression in Health and Disease

CSPG4 is heterogeneously expressed in stem-cells and adult progenitor cells [11] such as epidermal stem cells [12], naive and (to a greater degree) activated pericytes—the latter being associated with wound healing and a range of fibrotic pathologies [13,14]. In normal adult tissues, expression appears to be post-translationally downregulated at terminal differentiation [6,15], while elevated expression in adult tissues is associated with poorly-differentiated and EMT-characterized cells with tumor forming potential [5,9]. Upregulation of CSPG4 in malignant tumors is putatively linked to their ability to bypass inherent regulatory mechanisms by suppressing apoptotic signaling, proliferating at an elevated rate and acquiring anchorage independence. This is evident from the observation that treatment with an anti-CSPG4 monoclonal antibody (mAb) results in reduced growth and motility of melanoma cells [16].

CSPG4 is becoming increasingly implicated in several of the most aggressive and treatment-resistant forms of cancer, including malignant melanoma [3,17], basal-like breast cancers, leukemia, mesothelioma, glioblastoma multiforme, soft-tissue sarcomas, pancreatic carcinoma and squamous cell carcinoma of the head and neck (HNSCC) [4,18,19,20,21,22,23,24,25]. CSPG4 overexpression is particularly associated with mid-to-late transformation stages, from proliferative to early invasive and migratory stages of tumor progression [3,9], such as in the progression of melanoma tumors from radial growth phase (RGP) to vertical growth phase (VGP).

Of particular interest is the fact that CSPG4 is also putatively expressed by cancer stem cells (CSCs) [4]. CSCs are poorly differentiated, self-renewing cancer cells with highly regenerative, migratory and tumor forming ability [23,24,25]. CSCs represent a small but distinct subpopulation within tumors that are particularly resistant to several modes of cytotoxic therapy. This resilient phenotype allows CSCs to re-establish local and/or distal tumors even after seemingly complete eradication of tumors by conventional therapy [4,26]. This feature furthers the value of CSPG4 expression in cancer as a prognostic marker, particularly pertaining to metastasis, treatment resistance and probability of relapse.

CSPG4 possesses no known catalytic function of its own, however it is able to facilitate constitutive activation of signaling pathways which promote cell proliferation, migration and invasion [9]. Via various binding sites dispersed in its large extracellular domain, CSPG4 interacts with several ligands, such as growth factors, in the extracellular space. For example, fibroblast growth factor (FGF) and platelet-derived growth factor AA (PDGF-AA) associate with CSPG4, which in turn mediates binding to their respective receptors and protects their protease-sensitive basic Lys and Asp/Arg-rich adhesion sites from extracellular degradation [15]. Consequently, the association of FGF and PDGF-AA with CSPG4 promotes their activation of the intracellular Ras-RAF-MEK-MAPK and PI-3K/AKT/mTOR pathways, which enhance gene transcription, ultimately promoting cell proliferation and cell migration [26]. Moreover, together with melanoma cell adhesion molecule (MCAM), CSPG4 is also implicated as a mediator of PAX3 (of the paired box or PAX family) signaling. PAX3 is a developmentally important transcription factor which, in adults, is highly implicated in melanoma progression, promoting a poorly-differentiated and migratory phenotype [27].

Additionally, CSPG4 is known to cooperate with *integrins*, transmembrane proteins which interact with extracellular matrix (ECM) components such as collagen, fibrinogen, galectin-3, fibronectin, vascular cell adhesion molecule (VCAM1) and cell-surface syndecans [28]. CSPG4 acts as co-receptor with α_4_β_1_ integrin in associating with both fibronectin and VCAM1, triggering integrin clustering and fibronectin matrix assembly [8]. Galectin-3, a lectin family member strongly associated with tumor metastasis, has been shown to form a complex with α_4_β_1_ integrin and CSPG4 [6]. Clustering of CSPG4 and integrins also induces activation of the Rho family of GTPases [29], which regulate the formation of cytoskeletal extensions characteristic of migratory cells, such as filopodia, via phosphorylation and recruitment of focal adhesion kinase (FAK) and, further downstream, phosphorylation of p130^cas^, a docking protein credited for mediating anchorage independence [8,29,30]. Furthermore, CSPG4 has also been shown to bind (in a CSGAG dependent manner) to membrane type-3 matrix metalloproteinase (MT3-MMP) and its substrate proMMP-2, presenting proMMP-2 to MT3-MMP for activation resulting in downstream promotion of cell migration [31].

Taken together, CSPG4 is overexpressed in multiple difficult-to-treat cancers in which it is a prominent player in promoting tumor progression and metastasis via various modes of action. Practically, such a central oncogenic role may represent a druggable Achilles’ heel for rational-designed CSPG4-targeted cancer therapy.

For the purposes of this review, we recognize CSPG4 as a marker for aggressive, therapy-resistant cancers and are particularly interested in its potential as target for tumor-selective oncolytic agents such as antibody-drug conjugates (ADCs), immunotoxins (ITs) and tumor necrosis factor (TNF)-related apoptosis-inducing ligand (TRAIL) fusion proteins, described later in this review.

## 2. CSPG4 as Target for Cancer Therapy

Several mAb targeting CSPG4 have been described which inhibit growth and progression of CSPG4-positive tumors, including mAb 9.2.27 (against melanoma [16]), mAb 225.28 (against breast cancer [18]) and mAb TP41.2 (against mesothelioma [21]). The antitumor activity of anti-CSPG4 mAb 9.2.27 appeared to be largely attributable to its ability to block binding of the afore mentioned ECM-associated ligands to the extracellular domain of CSPG4 [16]. Similarly, the anti-CSPG4 mAb 225.28 potently suppressed proliferation and metastasis in CSPG4^+^ breast cancer cells by inhibiting CSPG4-mediated survival-promoting signaling pathways [18]. Moreover, Chang and co-workers provided supporting evidence for non-immunological mechanisms by which anti-CSPG4 mAbs affect the biology of melanoma cells [32]. Rivera and co-workers monitored the downstream signaling components involved in CSPG4-mediated ECM interactions and found that TP41.2 inhibited cell-adhesion dependent survival in malignant mesothelioma [21].

Therapy with conventional naked murine antibodies is known to have only limited anti-tumor activity in vivo. This may be explained by various mutually reinforcing negative factors, including high immunogenicity, reduced serum half-life and an inability to adequately recruit human cytotoxic effector mechanisms such complement dependent cytotoxicity (CDC) and antibody dependent cell-meditated cytotoxicity (ADCC). Furthermore, antibodies as such have relative high molecular weights and bulkiness that limit their penetration of solid tumors.

**Figure 2:**
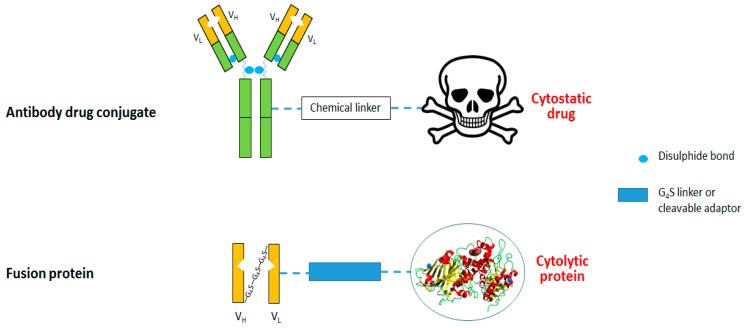
Immunotoxins comprising antibodies linked to an anti-cancer agent. The scFv portion (yellow frame) of the monoclonal antibody (green frame) retains antigen specificity and binding capacity. Antibody drug conjugate: 1st generation (monoclonal antibody) and 2nd generation (scFv), conjugated to a chemotherapeutic drug; Fusion protein: 3rd generation (bacterial/viral effector) and 4th generation (human effector). Recombinant proteins enable the introduction of a variety of adaptor peptides to facilitate cytosolic delivery (blue rectangle).

Antibody engineering has been applied to address these and other issues. This has led to the construction of novel CSPG4-targeted antibody-based agents with significantly enhanced anticancer activity. ITs represent a broad range of ADCs that enable selective delivery and subsequent internalization of cytotoxic drugs into tumor cells (Figure 2). ADCs can be generated by chemical conjugation of a toxic agent to a conventional antibody via a conditionally stable linker [33]. Ideally, this linker should remain fully intact until the ADC binds to the target cell, only releasing the toxic drug moiety upon internalization [33]. Recently, a novel biocompatible CSPG4-targeted ADC format, designated HFt-Pt-Ep1, was described in which up to three molecules of the anti-CSPG4 mAb Ep1 were conjugated to a single human ferritin (HFt) cage encapsulating up to 50 cisplatin molecules. Treatment of CSPG4^+^ cancer cells selectively and effectively inhibit thymidine incorporation with negligible effect on CSPG4-negative cell lines [34].

Antibody technology has been improved significantly by the advent of antibody-phage display libraries, which permits panning out of unique disease-specific antibody fragments for use as building blocks to construct fully functional antibodies and derivatives thereof [35]. This method has also enabled the selection of fully human single chain variable antibody fragments (scFv’s), some of which have been used to replace non-human antibody domains from conventional ADCs, thereby reducing the risk of immunogenicity when applied in humans.

Moreover, presumably due to smaller size, scFv antibodies may show improved tumor penetration compared to their mAb and Fab_2_ counterparts [36], while maintaining target tissue specificity. In the context of this review is worth mentioning that a human anti-CSPG4 antibody derivative, scFv-Fc21, has been shown to inhibit tumor growth and migration similarly to its full mAb counterpart [37].

## 3. Anti-CSPG4 Immunotoxins

Protein-based ITs consist of a disease-specific antibody fragment (targeting domain) recombinantly fused to a cytotoxic protein, such as Exotoxin A (effector domain) separated by intramolecular peptide linker [38]. In general, ITs exhibit acceptable tumor penetration and serum stability. Improved tumor penetration is achieved by using a tumor-targeting scFv (or nanobody) [36] and the smallest truncated form of a protein toxin that still retains its cytotoxicity. This approach yields a much smaller drug molecule than the first-generation full-length mAb and full-length toxin versions. Protein engineering technology has also been used to design ranges of peptide linker variants, including linkers with elements that facilitate production and purification, as well as those that improve serum stability and influence intracellular routing of the IT [39,40,41].

Exotoxin A (ETA) is a potent cytotoxic protein produced by *Pseudomonas aeruginosa*, ETA consists of three functional domains: a cell binding domain (I), an intracellular membrane translocation domain (II) and an adenosine diphosphate (ADP)-ribosylating domain (III) [42]. Domain III is responsible for the toxicity of ETA by inactivating elongation factor 2 (EF-2) via ADP-ribosylation, interrupting protein production and inducing cell death with great potency (theoretically, a single molecule ETA can induce full arrest of cellular biosynthesis). Already in the early stages of IT development, ETA was recognized as a cytotoxic effector domain with promising anticancer activity [43]. The intrinsic cell binding and translocation capacity of full-length ETA ensures cytosolic delivery of the toxic domain III. However, these same characteristics of ETA are also the source of tumor specificity problems, as non-specific cell binding leads to off-target toxicity. This problem was largely overcome by employing a truncated form of ETA (ETA′) which lacks its cell binding Domain Ia [44], such as used in an ETA′-based IT targeting MSCP (CSPG4) recently generated by Brehm and co-workers [21] using a novel bacterial expression system [45].

In the following sections, we will provide a short overview of CSPG4-targeting scFv-fusion proteins which have been preclinically evaluated for efficacy towards various refractory cancer types, in particular in rhabdomyosarcoma (RMS), a soft-tissue sarcoma occurring in young children (in the form of embryonal RMS, eRMS) as well as older children and adults (typically alveolar or pleomorphic RMS, aRMS or pRMS) [46]. The different forms of RMS have distinct molecular origins and can occur in a wide range of tissues, hence a combination of conventional therapeutic modalities is recommended for the treatment of these resilient neoplasms. Unfortunately, those treatments are associated with adverse long-term effects and shortened life-expectancy [47].

**Figure 3:**
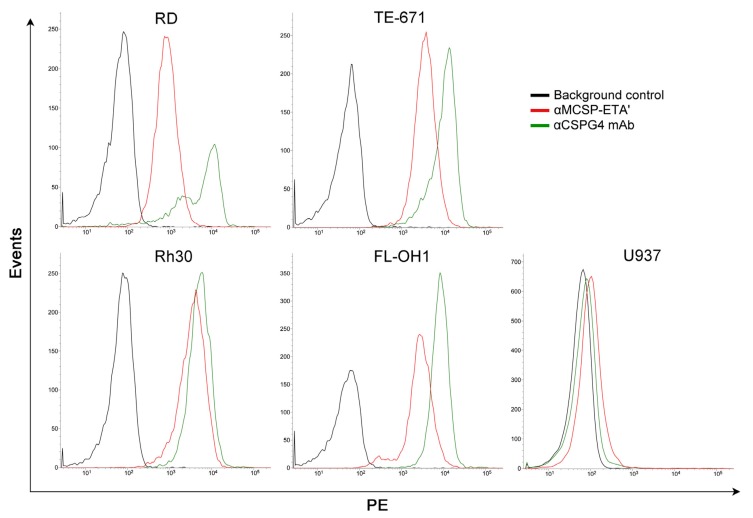
Binding analysis of αCSPG4-ETA′ (αMCSP-ETA′) immunotoxin to rhabdomyosarcoma (RMS) cell lines (RD, TE-671, Rh30 and FL-OH1) versus CSPG4^−^ U937 lymphoma cells. All cells lines were incubated with αCSPG4 mAb or αMCSP-ETA′ and analyzed by flow cytometry. Reprinted from Brehm et al. (2014) with permission from Elsevier [24].

Brehm and co-workers screened their αMCSP-ETA′ compound for differential binding and cytotoxic activity towards notoriously aggressive CSPG4^+^ eRMS and aRMS cell lines (RD, FL-OH1, TE-671 and Rh30) versus a CSPG4-negative lymphoma line (Figure 3). Preferential binding of αMCSP-ETA′ was detected in all RMS lines and in primary RMS patient-derived cancer cells. Moreover, upon binding rapid internalization of αMCSP-ETA′ was observed in all RMS cell lines, while no binding or internalization was observed for the CSPG4^−^ U937 lymphoma cells. Importantly, in vitro treatment with αMCSP-ETA′ inhibited cell proliferation and promoted apoptosis in all CSPG4^+^ lines tested at low nanomolar range, while sparing CSPG4^−^ cells. Additionally, a non-targeting mock IT (Mock-ETA′) did not have any effect on the MCSP^+^ RMS cells.

### 3.1. Anti-CSPG4 Human Cytolytic Fusion Proteins

As previously mentioned, despite their potent in vitro anticancer activity, ETA-based ITs have several limitations, including immunogenicity (due to their non-human nature) and off-target toxicities due to their inherent infectious nature. Truncated versions of ETA (ETA′) have been implemented, primarily retaining domains II (membrane translocation capacity) and domain III (ADP-ribosyltransferase activity) of the full virulence factor [48]. However, ETA′-based ITs still hold limited potential for clinical applications due to their immunogenicity in humans, an obstacle recently being addressed by the depletion of recognized B-cell epitopes on the IT [49]. In this respect, entirely-human ITs, in which both the targeting moiety and effector protein are of human origin, are representing a very attractive solution: recombinant human cytolytic fusion proteins (hCFPs) present a viable alternative, allowing repeated administrations without requiring compensatory dose increases.

Having established the specific anticancer activity of αMCSP-ETA′ in CSPG4^+^ RMS cell lines, Brehm and co-workers proceeded to develop and test pro-apoptotic human proteins as potential hCFP candidates [50] to replace ETA′, including the cytotoxic protein MAP tau.

Microtubule-associated protein tau (MAP tau) is most commonly associated with neurobiology research due to its implication in Alzheimer’s disease [51]. The timely polymerization and de-polymerization of microtubules is crucial for mitosis [52], a process which is highly upregulated in cancer cells. The rationale behind exploiting MAP tau as a cytotoxic effector domain is based on its ability to bind to and stabilize microtubule polymerization, disrupting mitosis and ultimately arresting cell division [53]. Employing MAP tau as a cytolytic effector has two major benefits: (a) it is a fully human apoptosis-inducing protein; and (b) it has no known endogenous inhibitors, which may reduce its efficacy as a cytolytic agent.

Amoury and co-workers generated a CSPG4-targeting hCFP by replacing ETA′ with MAP tau as cytolytic effector [54]. The resulting αCSPG4(scFv)-MAP tau was similarly evaluated for binding and internalization in CSPG4^+^ human triple-negative breast cancer (TNBC) cell lines, alongside CSPG4^−^ mammary carcinoma lines. TNBC is a highly aggressive and treatment-resistant breast cancer characterized by the absence/under-expression of three commonly targeted receptors: estrogen receptor (ER), progesterone receptor (PR) and human epidermal growth factor receptor II (HER2). This profile, also commonly seen in basal-like breast cancers, renders tumors resistant to endocrine-receptor targeted therapy, making non-surgical treatment of such subsets of breast cancer somewhat challenging. Furthermore, both basal and TNBC classifications are associated with the presence of cancer stem cell (CSC) populations, which are implicated in the renewal and therapy-resistance of several types of solid tumors [55]. As previously mentioned, CSPG4 has been identified as a marker both for CSCs [4,26] and for TNBC [15], providing further support of its potential as target antigen for hCFP therapy in TNBC.

**Figure 4:**
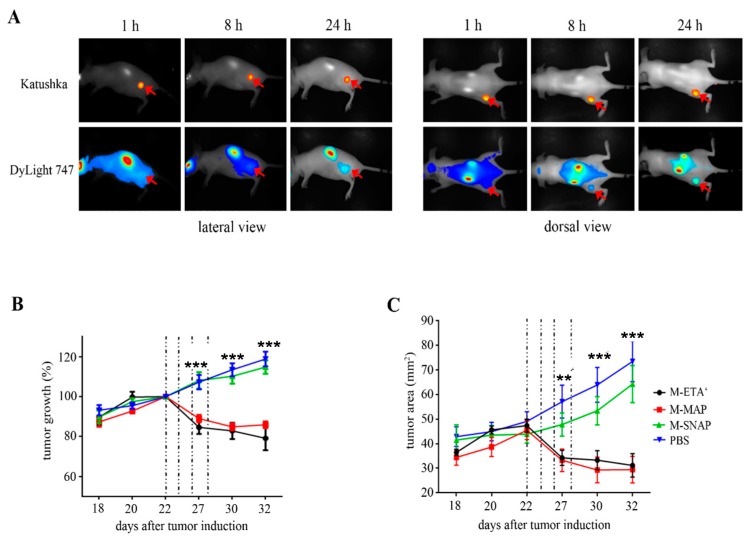
Treatment with αMCSP-MAP tau in mouse xenograft tumor model. (**A**) Distribution of αMCSP-MAP tau over time post-injection—distribution of DyLight 747-labeled hCFP (blue) in relation to Katushka-labeled (red) tumor tissue was monitored by whole body imaging. Red arrows indicate tumor site; (**B**) Relative tumor size of xenograft mice treated with αMCSP-MAP tau, αMCSP-ETA′, αMCSP-SNAP and phosphate buffered saline (PBS). Tumor size was measured using caliper and normalized to pre-treatment tumor size; (**C**) Tumor volume calculated from caliper measurements. *p*-Values of *** *p* ≤ 0.001 and ** *p* ≤ 0.005 were based on two-way ANOVA statistical analysis. Reprinted with permission [54]. Copyright 2016 International Journal of Cancer.

Indeed, an αCSPG4(scFv)-MAP tau exhibited specific binding to CSPG4^+^ TNBC lines MDA-MB-231 and Hs578T, with no binding to CSPG4^−^ MDA-MB-468 cells. Importantly, internalization of αCSPG4(scFv)-MAP tau was demonstrated to be CSPG4-dependent. Nuclear-localized αCSPG4(scFv)-MAP tau interacted with tubulin and promoted polymerization, as shown by means of co-immunoprecipitation and polymerization assays. Although less potent than CSPG4-ETA′, αCSPG4(scFv)-MAP tau induced a partial G2/M arrest and eventually apoptosis in CSPG4^+^ cells. Studies in tumor-xenografted mice demonstrated effective suppression of TNBC tumors, whereas no effect was detected when treated with the MOCK control fusion protein αCSPG4(scFv)-SNAP. In addition, the in vivo toxicity of αCSPG4(scFv)-MAP tau was comparable to that of its ETA′-based counterpart for depletion of triple-negative breast cancer (TNBC) tumors (Figure 4). No detectable side effects were observed regardless of treatment dose of αCSPG4(scFv)-MAP tau. In contrast, severe detrimental effects were observed during αCSPG4-ETA′ treatment. Thus, αCSPG4(scFv)-MAP tau showed to be both selectively efficacious against an otherwise therapy-resistant cancer type, with little or no apparent systemic toxicity.

The benefits associated with humanization of ITs have led to further exploration into CSPG4-targeting hCFP development. Other candidates recently being investigated as suitable hCFP effector domains include Granzyme B (GrB), a serine protease secreted by cytotoxic lymphocytes [56] and angiogenin (Ang), a human ribonuclease with a wide range of roles in biosynthesis and stress-induced apoptosis [38,39,40]. The natural killer GrB, in fusion with a scFv, has been shown to effectively deplete hematological tumors [41,57] and is undergoing evaluation for treatment of solid tumors [56]. Ang, which induces apoptosis by degrading tRNA, has also been shown to exhibit cytotoxicity upon delivery via a disease-specific scFv, while producing no immunogenicity or off-target toxicity [38,43,58]. Both these cytotoxic effectors are subject to endogenous inhibition as a tumor survival mechanism and the most recent stages of proofing have involved modifications to reduce sensitivity to inhibition [44].

### 3.2. Targeted Anticancer Strategies Using CSPG4-Targeted Death Inducing TNF Ligands

Despite the many recent biotechnological advancements, antibody-based targeted approaches in oncology are still far from perfect. There are still many short-comings in the field of tumor-directed therapeutic protein drugs with highly potent anticancer activity, while yet retaining a safe toxicity profile. Ideally, therapeutic agents used for targeted approaches should remain systemically inactive and gain full anti-tumor activity only after selective binding to malignant target cells. In this respect, the immune system has been hailed for its stringent spatial and temporal regulation of its highly potent effector mechanisms. Cytotoxic T-cells, in particular, appear to be perfectly equipped for efficient and directed cancer cell destruction. After TCR-mediated specific recognition and appropriate co-stimulatory confirmation, efficient target cell elimination is achieved via membranolytic pro-apoptotic effector molecules like perforins and granzymes and/or via receptor-mediated apoptosis induction through so-called death receptors, like FAS, TNFR and TRAILR. Targeted anticancer strategies using these death-inducing ligands, such as TRAIL, therefore possess considerable appeal.

### 3.3. Soluble TRAIL for Cancer Therapy

**Figure 5:**
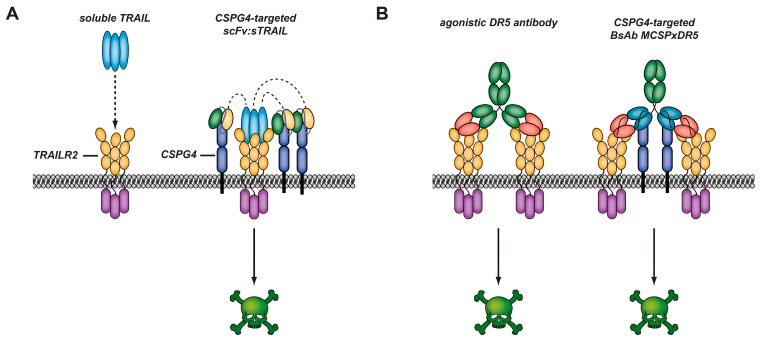
(**A**) Soluble TRAIL (sTRAIL, light blue) has limited pro-apoptotic capacity towards cancer cells as it fails to effectively cross-link TRAIL-R2 (DR5, yellow). ScFv:sTRAIL fusion proteins, consisting of sTRAIL recombinantly fused (as indicated by the dotted lines) to a scFv (heavy & light chains in green & beige) specific for a tumor-associated cell surface antigen such as CSPG4 (indigo), are designed to initiate cross-linking of agonistic TRAIL receptors only upon specific binding to the above-mentioned preselected antigen; (**B**) A conventional agonistic anti-DR5 antibody like tigatuzumab (green) may induce apoptosis in DR5-expressing cancer cells, but lacks tumor-selective binding activity. This may explain the limited efficacy observed for this antibody in clinical trials. In contrast, bsAb MCSPxDR5, which is composed of constant (green) and two serial variable regions (blue and red) with high specificity for both CSPG4 (indigo) and DR5 (yellow), resulting in agonistic activity towards DR5 as well as potent CSPG4-directed pro-apoptotic anticancer activity (solid arrows indicate agents which induce apoptosis).

TRAIL is expressed as a type II transmembrane protein on immune effector cells, whereas proteolytic processing or alternative splicing may yield soluble forms of TRAIL in vivo. Soluble forms of TRAIL appear to retain potent antitumor activity with no or minimal toxicity towards normal cells in numerous pre-clinical models. To take full advantage of TRAIL-based approaches, various characteristics of TRAILR/TRAIL system should be taken into account, particularly the ubiquitous expression of TRAIL receptors, the differential cross-linking requirements of agonistic receptors TRAIL-R1 (DR4) and TRAIL-R2 (DR5) and lastly, the solution behavior of certain sTRAIL preparations. These factors considered, an increasing number of researchers (some of the authors included) have constructed TRAIL fusion proteins, designated scFv:sTRAIL, comprising anticancer scFv antibody fragment genetically fused to the N-terminus of human soluble TRAIL (Figure 5).

### 3.4. CSPG4-Restricted Apoptosis Induction by αMCSP:sTRAIL

ScFv:sTRAIL fusion proteins are equipped with the capacity to activate agonistic TRAIL receptors after specific binding to a preselected cell surface-expressed tumor-associated antigen. Disease-specific apoptosis induction by scFv:sTRAIL fusion proteins has been demonstrated for various malignancies, including αMCSP:sTRAIL which targets melanoma featuring over-expression of CSPG4 [45]. Binding of αMCSP:sTRAIL converts this soluble and conditionally-inactive fusion protein into a membrane-bound form of TRAIL which is capable of signaling apoptosis not only through TRAIL-R1 but also through TRAIL-R2. Indeed, incubation of a series of melanoma cell lines with αMCSP:sTRAIL resulted in potent and MCSP-restricted apoptosis induction within 16 h. Treatment with αMCSP:sTRAIL was accompanied by a rapid dephosphorylation of Focal adhesion kinase (FAK), a kinase that has been implicated in the malignant phenotype of MCSP-expressing melanoma cells. Importantly, already at picomolar concentrations αMCSP:sTRAIL reduced anchorage-independent growth of melanoma cells by 50%. In contrast, in the same experiment an over 100-fold higher concentration of soluble TRAIL was unable to inhibit colony formation.

In xenograft mouse models with established A375M xenografted melanoma tumors, daily in vitro treatment with only 0.14 mg/kg/day αMCSP:sTRAIL resulted in a significant tumor growth retardation that was synergized when this treatment was combined with rimcazole, a σ-1 receptor antagonist that is currently evaluated in clinical trials for various cancers. 

Importantly, size-exclusion chromatography demonstrated that αMCSP:sTRAIL was produced as thermodynamically stable homogeneous trimers when excreted by eukaryotic production cells. Homotrimeric fusion proteins showed the expected molecular weight of ~180 kDa, which may prevent renal clearance likely resulting in an improved half-life of scFv:sTRAIL compared to conventional soluble TRAIL preparations.

Stable trimeric scFv:sTRAIL fusion proteins like αMCSP:sTRAIL contain 3 identical tumor targeting scFv domains. This trivalency may promotes accumulation of this fusion protein at the cell surface of MCSP-overexpressing tumor cells due to the associated avidity effect. Increased avidity is known to be beneficial for in vivo tumor targeting in many antibody-based therapeutic strategies [59].

Typically, on cancer cells the number target antigens like MCSP greatly exceed the number of TRAIL receptor molecules. Consequently, upon MCSP-directed binding of αMCSP:sTRAIL a net surplus of sTRAIL domains remains present to allow for subsequent activation of agonistic TRAIL receptors on neighboring tumor cells. This so-called bystander effect has the potential to also eliminate neighboring tumor cells that lack target antigen expression and which would otherwise escape from targeted therapy (Figure 3). Exceptional potent bystander of scFv:sTRAIL has been demonstrated both in vitro and in vivo for various malignancies [45,60,61,62]. Taken together, CSPG4-directed scFv:sTRAIL fusion proteins appear to be a favorable format for the targeted and safe elimination of cancer cells with heterogeneous expression of MCSP.

### 3.5. CSPG4-Restricted Apoptosis Induction by Bispecific Antibody MCSPxDR5

Pro-apoptotic activation of TRAIL-receptors for cancer therapy has been evaluated for both soluble forms of recombinant TRAIL and agonistic αTRAIL-receptor antibodies directed against DR4 or DR5. In particular, a series of agonistic αDR5 antibodies have been developed, including conatumumab, drozitumab, tigatuzumab and lexatumumab. Early phase clinical trials indicate good tolerability of these antibodies in patients, but also a lack of sufficient clinical efficacy. This appears to be related to the fact that these agonistic αDR5 antibodies have no tumor-selective binding activity themselves, whereas TRAIL receptors are ubiquitously expressed on normal tissue. As a result, relatively high concentrations of agonistic αDR5 antibodies maybe needed to overcome the target antigen sink formed by DR5 expressed on normal cells. Moreover, similar to sTRAIL, in order to gain therapeutic activity agonistic αDR5 antibodies such as tigatuzumab require secondary crosslinking e.g., by binding to FcχR receptors as present on myeloid effector cells.

As a result, a novel bispecific antibody-based approach was developed that promotes CSPG4-directed pro-apoptotic activation of DR5. This entailed engineering of a novel recombinant bispecific antibody, designated bsAb MCSPxDR5, that has both high binding affinity for the CSPG4 and potent agonistic activity towards DR5. The mode-of-action of bsAb MCSPxDR5 involved high-affinity binding to tumor cell surface-expressed CSPG4 with concomitant localized enhanced cross-linking of DR5. Herein, binding of bsAb MCSPxDR5 to tumor cells appeared to be dominated by binding to CSPG4.

The antitumor activity of tumor-bound bsAb MCSPxDR5 could be further enhanced by cross-linking of its human immunoglobulin G1 (IgG1) domain either by an artificial cross-linker or by Fc receptors as present on myeloid immune effector cells. Moreover, various melanoma-relevant drugs (bortezomib, valproic acid and vemurafenib) synergized the antitumor activity of bsAb MCSPxDR5. Importantly, MCSPxDR5 induced potent apoptosis in 11 out of 11 MCSP-expressing primary patient-derived melanoma cells, that was further enhanced after secondary cross-linking of its human IgG1 Fc domain. Of note, the fully functional human IgG1 Fc domain present in bsAb MCSPxDR5 allows for ADCC- and CDC-mediated elimination of cancer cells. Moreover, this Fc domain contained the binding motif for recycling by the neonatal Fc receptor (FcRn), which is of crucial importance for achieving appropriate half-lives in the circulation.

Current in vitro data indicate that MCSPxDR5 has potent CSGP4-directed pro-apoptotic activity towards MCSP^pos^/DR5^pos^ melanoma cells with essentially no or minimal toxicity towards various normal cells. Therefore, bsAb MCSPxDR5 may be of value for the targeted treatment of melanoma and other CSPG4-expressing malignancies. However, more in-depth studies are needed to establish appropriate efficacy and safe toxicity profile when applied in eligible cancer patients.

## 4. Discussion

While chemotherapy remains one of the most crucial treatment routes for cancer patients, it often comes up against obstacles such as resistance (upregulation of tumor survival pathways), recurrence (due to endurance of tumor subpopulations) and harmful side effects (due to lack of drug specificity). Tumors that do not express the more common characteristic biomarkers (e.g., endocrine receptors) are resistant to existing chemotherapies. The understanding that tumors must upregulate the expression of certain signaling proteins in order to meet their metabolic needs, provides us with a plethora of tumor-associated antigens which exhibit differential expression in otherwise receptor-negative cancers. Here we have described CSPG4, a trans-membrane protein involved in numerous pro-oncogenic signaling pathways, as a target for recombinant immunotherapeutics in aggressive, therapy-resistant cancers. CSPG4 is strongly associated with several hallmarks of advanced cancer, including proliferation, invasion and metastasis. It is known to be overexpressed in several types of melanoma, basal-like breast and several other highly aggressive cancers. Overexpression of CSPG4 has been associated with hypomethylation of CpG islands in the promoter region [10], while others have postulated that chromosomal translocations at 11q23, in a gene possibly encoding a transcription factor that regulates CSPG4 transcription, may result in CSPG4 overexpression (based on studies of patients with acute myeloid/lymphoblastic leukemia) [19,20].

The association of CSPG4 with typically treatment-resistant cancers and its putative overexpression in CSCs qualify it as an ideal target for immunotherapy. Treatment of melanoma, TNBC and mesothelioma with mAb 9.2.27 [16], mAb 225.28 [18] and TP41.2 [21], respectively, has been shown to suppress CSPG4-mediated cancer progression. However, the antigen-specificity of immunotherapy, including treatment with disease-specific ITs (the primary focus of this review), begs the consideration of potential detrimental side-effects of CSPG4-targeted therapy on non-malignant CSPG4^+^ progenitor cells, glial stem cells and pericytes.

Naive pericytes, which provide structural support to normal vasculature, express relatively low levels of CSPG4, whereas activated pericytes, mobilized during wound healing and tumor angiogenesis [14], express higher levels of CSPG4 [4]. A slight growth decrease in CSPG4^−^ melanoma tumors (in a mouse model exposed to anti-CSPG4 immunotherapy) was observed by Wang et al. [4], suggesting that the depletion of activated pericytes may suppress angiogenesis (a crucial feature of tumors) while also reducing the number of viable pericytes lining blood vesicles which may sensitize tumors to penetrating therapeutic agents. This and previous studies appear to imply that collateral toxicity to pericytes may not be detrimentally harmful to the patient and may in fact enhance tumor-eradication by ITs.

A similar outcome has been predicted for activated macrophages/microglia, which express NG2 (based on studies in mouse and rat brains). Systemic insult, such as tissue damage and neoplasm regeneration (such as following radiation therapy), induces the recruitment of activated macrophages to the site to neutralize the threat. However, factors present in the tumor microenvironment have been known to reprogram macrophages to cease their attack on the tumor [63]. These tumor-associated macrophages (taMΦ), including microglia recruited during CNS regeneration, proceed to secrete factors that promote tumor survival and progression. NG2 expression is upregulated in taMΦ [55] with respect to normal macrophages, which may target them for eradication at lower serum concentrations of ITs than naïve macrophages.

No adverse side effects have as yet been reported in immunotherapeutic studies targeting CSPG4 in multiple cancers [18,54,64], with the exception of potential immunogenicity caused by pathogenic cytotoxic effectors. However, in vivo studies are typically short-to-medium term mouse trials and do not provide information on the long-term impact on development or immunity. The conclusion may lie in differential expression: since NG2/CSPG4 have multiple tumor-promoting influence, the benefits of targeting cells which have upregulated CSPG4 may overshadow any non-tumor specific binding.

An anti-CSPG4(scFv) armed with a bacterial virulence factor has exhibited promising anticancer activity in RMS (Brehm et al.) [24]. However, although virulence factors are very potent cytotoxic effectors, their native binding and penetrative abilities tend to produce off-target effects and collateral toxicity. Deletion of the cell-binding domain I (such as in the case of MCSP-ETA′) does alleviate this issue by rendering the IT reliant on its targeting moiety (scFv) for binding and internalization, thereby reducing off-target binding. To further complicate matters, as non-human proteins bacterial toxins are inclined to induce production of neutralizing antibodies which sequester and inactivate the drug, thus limiting the number of treatment cycles that can be effectively administered [65]. This immunogenic effect can be somewhat reduced by identifying B-cell epitopes within the fusion protein and depleting them by mutagenesis [49].

In the interest of enhancing prolonged drug efficacy, complete elimination of immunogenicity and off-target toxicity can be achieved by replacing the bacterial toxin with an apoptosis-inducing human protein [38]. Recently, hCFPs have been shown to exhibit anticancer activity inTNBC [54], RMS [50,56] and leukemia [40,57], while notably no immunogenic effects were observed in these studies.

While reducing immunogenicity and off-target activity, exchanging a bacterial toxin for a less immunogenic human effector protein introduces additional obstacles that limit the potency of hCFPs. Bacterial toxins, once inside the cell, can translocate through subcellular membranes to access their subcellular target compartment, where they produce high toxic potency—a feature lacking in human proteins. Several strategies have been described by which it may be possible to enhance the antitumor efficacy of hCFPs in order to match (or even exceed) that of bacterial ITs, while minimizing off-target toxicity [66]. Some of the key obstacles and associated improvements are described below:

Internalization typically requires cross-linking of multiple ligand-bound receptors (or antibody-bound antigens) to activate clathrin recruitment and induce receptor-mediated endocytosis [67]. Binding of multiple receptors can be achieved by modifying the length (and thereby rigidity) of the linkers between V_H_ and V_L_ regions to induce formation of bi- and trivalent scFv’s (described in detail in [68]). Monovalent ITs, such as those recently published using targeting CSPG4, are poorly internalized into target cells in comparison to the multivalent scFv [69,70].

Subcellular routing determines the delivery of the hCFP to its effective target compartment. Internalized proteins remaining in the endosome are doomed to lysosomal degradation, hence effective drug deployment requires endosomal escape, re-routing & translocation of the hCFP within the cell. These requirements could potentially be approached by including hydrophobic and cleavable elements in the hCFP architecture (reviewed in [71]). The translocation domains of virulence factors (such as ETA, ricin A and DT) can be mimicked in a recombinant human protein in order to (a) promote retrograde transport from endosome to the ER by introduction of a C-terminal KDEL signal and (b) facilitate membrane translocation from the ER into to the target compartment (s) by means of selectively-exposed translocatable domains. The latter forms part of an adaptor which links the scFv to the effector protein, and consists (in its simplest form) of an endosomal cleavable domain adjacent to a membrane translocation peptide [72], resulting in removal of the scFv post-internalization and cytosolic delivery of the smallest effective fraction of the hCFP.

Enzymatic activity may be influenced via various methods involving modification of the cytolytic effector peptide in order to reduce its sensitivity to endogenous inhibitors and/or boost its affinity for catalytic substrates [73,74]. Granzyme B (R201K) [44,57] and angiogenin (G85R/G86R and Q117G) [39,40] are examples pro-apoptotic proteins which have undergone such modifications to enhance their cytolytic activity. Further modifications of angiogenin are currently undergoing cell model screening in fusion with αCSPG4 (scFv) for improved cytolytic efficacy against several cancers.

Somewhat impervious to most of the above-mentioned obstacles are CSPG4-directed TRAIL fusions proteins, which need only contact the TRAIL death receptor in order to induce apoptosis The αCSPG4:sTRAIL fusion protein is a fully-human tumor-directed anticancer agent which exacts potent pro-apoptotic activity when bound to the cell surface of a malignant cell. Importantly, off-target binding may not result in undesirable cytotoxic effects, since normal cells appear to be largely resistant to TRAIL-induced apoptosis. Therapeutic activity of scFv:sTRAIL is independent of both internalization and intracellular routing, although internalization does not appear to inhibit its activity. Moreover, the anticancer activity of scFv:sTRAIL fusion protein synergizes with the activity of a large variety of clinically used anticancer agents, as well as with numerous agents under preclinical investigation.

Finally, therapeutic CSPG4-directed cancer cell death may also be achieved using a bsAb-based approach as is exemplified by bsAb MCSPxDR5 that can exert strong and selective DR5-dependent cytotoxic activity against CSPG4-expressing melanoma cells. Crosslinking of the antibody with Fcγ-receptorsincreased the cytotoxic potential further, without compromising its selectivity. This approach may offer an immunotherapeutic approach that combines several mutually reinforcing anticancer activities, including delivering the death receptor agonist to the malignant cell population, potent activation of DR5-mediated cell death signaling, and recruitment of Fcγ-receptor-carrying immune cells that can mount an immune response against the tumor cells.

## 5. Conclusions

Cancer, as a group of diseases sharing molecular similarities but highly variable prognoses, poses a massive socio-economic drain, necessitating improved therapeutic strategies with increased potency and reduced side effects. CSPG4 represents a highly useful marker of aggressive and motile cancers, thus offering an excellent opportunity to target cancers that are notoriously resistant to conventional therapies. In this respect, hCFPs, TRAIL fusion proteins and DR5-agonistic bispecific antibodies targeted at CSPG4 are highly promising candidates for the treatment of highly aggressive cancers with a high recurrence rate and poor prognosis.

## Supplementary Materials

Supplementary File 1

## References

[1] Stegmüller J., Schneider S., Hellwig A., Garwood J., Trotter J. (2002). AN2, the mouse homologue of NG2, is a surface antigen on glial precursor cells implicated in control of cell migration. J. Neurocytol..

[2] Campoli M.R., Chang C.-C., Kageshita T., Wang X., McCarthy J.B., Ferrone S. (2004). Human high molecular weight-melanoma-associated antigen (HMW-MAA): A melanoma cell surface chondroitin sulfate proteoglycan (MSCP) with biological and clinical significance. Crit. Rev. Immunol..

[3] Price M.A., Colvin Wanshura L.E., Yang J., Carlson J., Xiang B., Li G., Ferrone S., Dudek A.Z., Turley E.A., McCarthy J.B. (2011). CSPG4, a potential therapeutic target, facilitates malignant progression of melanoma. Pigment Cell Melanoma Res..

[4] Wang X., Wang Y., Yu L., Sakakura K., Visus C., Schwab J., Ferrone C., Favoino E., Koya Y., Campoli M. (2010). CSPG4 in cancer: Multiple roles. Curr. Mol. Med..

[5] Nicolosi P.A., Dallatomasina A., Perris R. (2015). Theranostic impact of NG2/CSPG4 proteoglycan in cancer. Theranostics.

[6] Wen Y., Makagiansar I.T., Fukushi J., Liu F.T., Fukuda M.N., Stallcup W.B. (2006). Molecular basis of interaction between NG2 proteoglycan and galectin-3. J. Cell. Biochem..

[7] Makagiansar I.T., Williams S., Mustelin T., Stallcup W.B. (2007). Differential phosphorylation of NG2 proteoglycan by ERK and PKCα helps balance cell proliferation and migration. J. Cell Biol..

[8] Yang J., Price M.A., Neudauer C.L., Wilson C., Ferrone S., Xia H., Iida J., Simpson M.A., McCarthy J.B. (2004). Melanoma chondroitin sulfate proteoglycan enhances FAK and ERK activation by distinct mechanisms. J. Cell Biol..

[9] Yang J., Price M.A., Li G.Y., Bar-Eli M., Salgia R., Jagedeeswaran R., Carlson J.H., Ferrone S., Turley E.A., McCarthy J.B. (2009). Melanoma proteoglycan modifies gene expression to stimulate tumor cell motility, growth, and epithelial-to-mesenchymal transition. Cancer Res..

[10] Warta R., Herold-Mende C., Chaisaingmongkol J., Popanda O., Mock A., Mogler C., Osswald F., Herpel E., Küstner S., Eckstein V. (2014). Reduced promoter methylation and increased expression of CSPG4 negatively influences survival of HNSCC patients. Int. J. Cancer.

[11] Campoli M., Ferrone S., Wang X. (2010). Functional and clinical relevance of chondroitin sulfate proteoglycan 4. Adv. Cancer Res..

[12] Legg J., Jensen U.B., Broad S., Leigh I., Watt F.M. (2003). Role of melanoma chondroitin sulphate proteoglycan in patterning stem cells in human interfollicular epidermis. Development.

[13] Schlingemann R., Rietveld F., de Waal R., Ferrone S., Ruiter D.J. (1990). Expression of the high molecular weight melanoma-associated antigen by pericytes during angiogenesis in tumors and in healing wounds. Am. J. Pathol..

[14] Fligny C., Duffield J.S. (2013). Activation of pericytes: Recent insights into kidney fibrosis and microvascular rarefaction. Curr. Opin. Rheumatol..

[15] Goretzki L., Burg M.A., Grako K.A., Stallcup W.B. (1999). High-affinity binding of basic fibroblast growth factor and platelet-derived growth factor-AA to the core protein of the NG2 proteoglycan. J. Biol. Chem..

[16] Harper J., Reisfeld R. (1983). Inhibition of anchorage-independent growth of human melanoma cells by a monoclonal antibody to a chondroitin sulfate proteoglycan. J. Natl. Cancer Inst..

[17] Kageshita T., Nakamura T., Yamada M., Kuriya N., Arao T., Ferrone S. (1991). Differential expression of melanoma associated antigens in acral lentiginous melanoma and in nodular melanoma lesions. Cancer Res..

[18] Wang X., Osada T., Wang Y., Yu L., Sakakura K., Katayama A., McCarthy J.B., Brufsky A., Chivukula M., Khoury T. (2010). Cspg4 protein as a new target for the antibody-based immunotherapy of triple-negative breast cancer. J. Natl. Cancer Inst..

[19] Smith F.O., Rauch C., Williams D.E., March C.J., Arthur D., Hilden J., Lampkin B.C., Buckley J.D., Buckley C.V., Woods W.G. (1996). The human homologue of rat NG2, a chondroitin sulfate proteoglycan, is not expressed on the cell surface of normal hematopoietic cells but is expressed by acute myeloid leukemia blasts from poor-prognosis patients with abnormalities of chromosome band 11q23. Blood.

[20] Behm F.G., Smith F., Raimondi S., Pui C., Bernstein I. (1996). Human homologue of the rat chondroitin sulfate proteoglycan, ng2, detected by monoclonal antibody 7.1, identifies childhood acute lymphoblastic leukemias with t (4; 11)(q21; q23) or t (11; 19)(q23; p13) and mll gene rearrangements. Blood.

[21] Rivera Z., Ferrone S., Wang X., Jube S., Yang H., Pass H.I., Kanodia S., Gaudino G., Carbone M. (2012). CSPG4 as a target of antibody-based immunotherapy for malignant mesothelioma. Clin. Cancer Res..

[22] Wang J., Svendsen A., Kmiecik J., Immervoll H., Skaftnesmo K.O., Planagumà J., Reed R.K., Bjerkvig R., Miletic H., Enger P.Ø. (2011). Targeting the NG2/CSPG4 proteoglycan retards tumour growth and angiogenesis in preclinical models of GBM and melanoma. PLoS ONE.

[23] Svendsen A., Verhoeff J.J., Immervoll H., Brøgger J.C., Kmiecik J., Poli A., Netland I.A., Prestegarden L., Planaguma J., Torsvik A. (2011). Expression of the progenitor marker NG2/CSPG4 predicts poor survival and resistance to ionising radiation in glioblastoma. Acta Neuropathol..

[24] Brehm H., Niesen J., Mladenov R., Stein C., Pardo A., Fey G., Helfrich W., Fischer R., Gattenlohner S., Barth S. (2014). A CSPG4-specific immunotoxin kills rhabdomyosarcoma cells and binds to primary tumor tissues. Cancer Lett..

[25] Jamil N.S., Azfer A., Worrell H., Salter D.M. (2016). Functional roles of CSPG4/NG2 in chondrosarcoma. Int. J. Exp. Pathol..

[26] Hardy K.M., Yatskievych T.A., Konieczka J., Bobbs A.S., Antin P.B. (2011). FGF signalling through RAS/MAPK and PI3K pathways regulates cell movement and gene expression in the chicken primitive streak without affecting e-cadherin expression. BMC Dev. Biol..

[27] Medic S., Rizos H., Ziman M. (2011). Differential PAX3 functions in normal skin melanocytes and melanoma cells. Biochem. Biophys. Res. Commun..

[28] Desgrosellier J.S., Cheresh D.A. (2010). Integrins in cancer: Biological implications and therapeutic opportunities. Nat. Rev. Cancer.

[29] Eisenmann K.M. (2000). Melanoma Chondroitin Sulfate Proteoglycan Stimulates Signal Transduction Pathways Associated with Cytoskeletal Reorganization and Tumor Cell Survival. Ph.D. Thesis.

[30] Hecker T.P., Gladson C.L. (2003). Focal adhesion kinase in cancer. Front. Biosci..

[31] Iida J., Wilhelmson K.L., Ng J., Lee P., Morrison C., Tam E., Overall C.M., McCarthy J.B. (2007). Cell surface chondroitin sulfate glycosaminoglycan in melanoma: Role in the activation of pro-MMP-2 (pro-gelatinase a). Biochem. J..

[32] Chang C.C., Campoli M., Luo W., Zhao W., Zaenker K.S., Ferrone S. (2004). Immunotherapy of melanoma targeting human high molecular weight melanoma-associated antigen: Potential role of nonimmunological mechanisms. Ann. N. Y. Acad. Sci..

[33] Alley S.C., Okeley N.M., Senter P.D. (2010). Antibody-drug conjugates: Targeted drug delivery for cancer. Curr. Opin. Chem. Biol..

[34] Falvo E., Tremante E., Fraioli R., Leonetti C., Zamparelli C., Boffi A., Morea V., Ceci P., Giacomini P. (2013). Antibody-drug conjugates: Targeting melanoma with cisplatin encapsulated in protein-cage nanoparticles based on human ferritin. Nanoscale.

[35] Ten Haaf A., Pscherer S., Fries K., Barth S., Gattenlöhner S., Tur M.K. (2015). Phage display-based on-slide selection of tumor-specific antibodies on formalin-fixed paraffin-embedded human tissue biopsies. Immunol. Lett..

[36] Firer M.A., Gellerman G. (2012). Targeted drug delivery for cancer therapy: The other side of antibodies. J. Hematol. Oncol..

[37] Wang X., Katayama A., Wang Y., Yu L., Favoino E., Sakakura K., Favole A., Tsuchikawa T., Silver S., Watkins S.C. (2011). Functional characterization of an SCFV-FC antibody that immunotherapeutically targets the common cancer cell surface proteoglycan CSPG4. Cancer Res..

[38] Rybak S.M., Hoogenboom H.R., Meade H.M., Raus J., Schwartz D., Youle R.J. (1992). Humanization of immunotoxins. Proc. Natl. Acad. Sci. USA.

[39] Cremer C., Vierbuchen T., Hein L., Fischer R., Barth S., Nachreiner T. (2015). Angiogenin mutants as novel effector molecules for the generation of fusion proteins with increased cytotoxic potential. J. Immunother..

[40] Cremer C., Braun H., Mladenov R., Schenke L., Cong X., Jost E., Brummendorf T.H., Fischer R., Carloni P., Barth S. (2015). Novel angiogenin mutants with increased cytotoxicity enhance the depletion of pro-inflammatory macrophages and leukemia cells ex vivo. Cancer Immunol. Immunother..

[41] Stahnke B., Thepen T., Stocker M., Rosinke R., Jost E., Fischer R., Tur M.K., Barth S. (2008). Granzyme b-H22(SCFV), a human immunotoxin targeting CD64 in acute myeloid leukemia of monocytic subtypes. Mol. Cancer Ther..

[42] FitzGerald D.J., Willingham M.C., Pastan I. (1988). Pseudomonas Exotoxin—Immunotoxins. Immunotoxins.

[43] Rybak S.M., Newton D.L. (1999). Natural and engineered cytotoxic ribonucleases: Therapeutic potential. Exp. Cell Res..

[44] Cremer C., Hehmann-Titt G., Schiffer S., Melmer G., Carloni P., Barth S., Nachreiner T. (2015). Engineered versions of granzyme b and angiogenin overcome intrinsic resistance to apoptosis mediated by human cytolytic fusion proteins. Resistance to Immunotoxins in Cancer Therapy.

[45] De Bruyn M., Rybczynska A.A., Wei Y., Schwenkert M., Fey G.H., Dierckx R.A., van Waarde A., Helfrich W., Bremer E. (2010). Melanoma-associated chondroitin sulfate proteoglycan (MCSP)-targeted delivery of soluble trail potently inhibits melanoma outgrowth in vitro and in vivo. Mol. Cancer.

[46] Parham D.M., Ellison D.A. (2006). Rhabdomyosarcomas in adults and children: An update. Arch. Pathol. Lab. Med..

[47] Stevens M.C. (2005). Treatment for childhood rhabdomyosarcoma: The cost of cure. Lancet Oncol..

[48] Weldon J.E., Pastan I. (2011). A guide to taming a toxin—Recombinant immunotoxins constructed from pseudomonas exotoxin a for the treatment of cancer. FEBS J..

[49] Nagata S., Pastan I. (2009). Removal of b cell epitopes as a practical approach for reducing the immunogenicity of foreign protein-based therapeutics. Adv. Drug Deliv. Rev..

[50] Brehm H., Hristodorov D., Pardo A., Mladenov R., Niesen J., Fischer R., Tur M.K., Barth S. (2015). Targeted killing of rhabdomyosarcoma cells by a map-based human cytolytic fusion protein. Cancer Lett..

[51] Fasulo L., Ugolini G., Visintin M., Bradbury A., Brancolini C., Verzillo V., Novak M., Cattaneo A. (2000). The neuronal microtubule-associated protein tau is a substrate for caspase-3 and an effector of apoptosis. J. Neurochem..

[52] Mandelkow E., Mandelkow E.-M. (1995). Microtubules and microtubule-associated proteins. Curr. Opin. Cell Biol..

[53] Hristodorov D., Mladenov R., Pardo A., Pham A.T., Huhn M., Fischer R., Thepen T., Barth S. (2013). Microtubule-associated protein tau facilitates the targeted killing of proliferating cancer cells in vitro and in a xenograft mouse tumour model in vivo. Br. J. Cancer.

[54] Amoury M., Mladenov R., Nachreiner T., Pham A.T., Hristodorov D., di Fiore S., Helfrich W., Pardo A., Fey G., Schwenkert M. (2016). A novel approach for targeted elimination of CSPG4-positive triple-negative breast cancer cells using a map tau-based fusion protein. Int. J. Cancer.

[55] Bu J., Akhtar N., Nishiyama A. (2001). Transient expression of the NG2 proteoglycan by a subpopulation of activated macrophages in an excitotoxic hippocampal lesion. Glia.

[56] Niesen J., Hehmann-Titt G., Woitok M., Fendel R., Barth S., Fischer R., Stein C. (2016). A novel fully-human cytolytic fusion protein based on granzyme b shows in vitro cytotoxicity and ex vivo binding to solid tumors overexpressing the epidermal growth factor receptor. Cancer Lett..

[57] Schiffer S., Hansen H.P., Hehmann-Titt G., Huhn M., Fischer R., Barth S., Thepen T. (2013). Efficacy of an adapted granzyme b-based anti-CD30 cytolytic fusion protein against pi-9-positive classical hodgkin lymphoma cells in a murine model. Blood Cancer J..

[58] Huhn M., Sasse S., Tur M.K., Matthey B., Schinköthe T., Rybak S.M., Barth S., Engert A. (2001). Human angiogenin fused to human CD30 ligand (ANG-CD30l) exhibits specific cytotoxicity against CD30-positive lymphoma. Cancer Res..

[59] Power B.E., Kortt A.A., Hudson P.J. (2003). Generation of recombinant multimeric antibody fragments for tumor diagnosis and therapy. Recomb. Antib. Cancer Ther..

[60] Bremer E., Samplonius D., Kroesen B.-J., van Genne L., de Leij L., Helfrich W. (2004). Exceptionally potent anti-tumor bystander activity of an SCFV: Strail fusion protein with specificity for EGP2 toward target antigen-negative tumor cells. Neoplasia.

[61] Bremer E., Samplonius D.F., Peipp M., van Genne L., Kroesen B.-J., Fey G.H., Gramatzki M., de Leij L.F., Helfrich W. (2005). Target cell-restricted apoptosis induction of acute leukemic T cells by a recombinant tumor necrosis factor–related apoptosis-inducing ligand fusion protein with specificity for human CD7. Cancer Res..

[62] Stieglmaier J., Bremer E., Kellner C., Liebig T.M., Ten Cate B., Peipp M., Schulze-Koops H., Pfeiffer M., Bühring H.-J., Greil J. (2008). Selective induction of apoptosis in leukemic b-lymphoid cells by a CD19-specific trail fusion protein. Cancer Immunol. Immunother..

[63] Hambardzumyan D., Gutmann D.H., Kettenmann H. (2016). The role of microglia and macrophages in glioma maintenance and progression. Nat. Neurosci..

[64] Mittelman A., Chen Z.J., Yang H., Wong G.Y., Ferrone S. (1992). Human high molecular weight melanoma-associated antigen (HMW-MAA) mimicry by mouse anti-idiotypic monoclonal antibody MK2–23: Induction of humoral anti-hmw-maa immunity and prolongation of survival in patients with stage iv melanoma. Proc. Natl. Acad. Sci. USA.

[65] Mazor R., Onda M., Pastan I. (2016). Immunogenicity of therapeutic recombinant immunotoxins. Immunol. Rev..

[66] Hetzel C., Bachran C., Tur M.K., Fuchs H., Stocker M. (2009). Improved immunotoxins with novel functional elements. Curr. Pharm. Des..

[67] Brown V.I., GREENE M.I. (1991). Molecular and cellular mechanisms of receptor-mediated endocytosis. DNA Cell Biol..

[68] Kortt A.A., Dolezal O., Power B.E., Hudson P.J. (2001). Dimeric and trimeric antibodies: High avidity scfvs for cancer targeting. Biomol. Eng..

[69] Le Gall F., Kipriyanov S.M., Moldenhauer G., Little M. (1999). Di-, tri-and tetrameric single chain fv antibody fragments against human CD19: Effect of valency on cell binding. FEBS Lett..

[70] Ribbert T., Thepen T., Tur M., Fischer R., Huhn M., Barth S. (2010). Recombinant, ETA′-based CD64 immunotoxins: Improved efficacy by increased valency, both in vitro and in vivo in a chronic cutaneous inflammation model in human CD64 transgenic mice. Br. J. Dermatol..

[71] Fuchs H., Weng A., Gilabert-Oriol R. (2016). Augmenting the efficacy of immunotoxins and other targeted protein toxins by endosomal escape enhancers. Toxins (Basel).

[72] Hetzel C., Bachran C., Fischer R., Fuchs H., Barth S., Stöcker M. (2008). Small cleavable adapters enhance the specific cytotoxicity of a humanized immunotoxin directed against CD64-positive cells. J. Immunother..

[73] Hehmann-Titt G., Schiffer S., Berges N., Melmer G., Barth S. (2013). Improving the therapeutic potential of human granzyme b for targeted cancer therapy. Antibodies.

[74] Cong X., Cremer C., Nachreiner T., Barth S., Carloni P. (2016). Engineered human angiogenin mutations in the placental ribonuclease inhibitor complex for anticancer therapy: Insights from enhanced sampling simulations. Protein Sci..

