# Supplementary material for: CSPG4: A Target for Selective Delivery of Human Cytolytic Fusion Proteins and TRAIL
Source: Biomedicines. 2017 Jun 28;5(3):37. doi: 10.3390/biomedicines5030037 (PMC5618295; doi:10.3390/biomedicines5030037)
Supplement: Supplementary File 1 [file biomedicines-05-00037-s001.pdf]

Copyright Clearance Center RightsLink®

Home | Account Info | Help

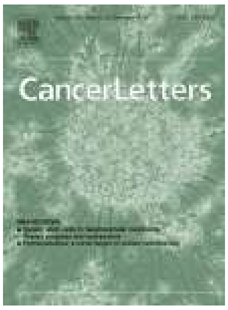

Title: A CSPG4-specific immunotoxin kills rhabdomyosarcoma cells and binds to primary tumor tissues

Author: Hannes Brehm,Judith Niesen,Radoslav Mladenov,Christoph Stein,Alessa Pardo,Georg Fey,Wijnand Helfrich,Rainer Fischer,Stefan Gattenlöhner,Stefan Barth

Publication: Cancer Letters

Publisher: Elsevier

Date: 1 October 2014

Copyright © 2014 Elsevier Ireland Ltd. All rights reserved.

Logged in as:
Stefan Barth
University of Cape Town

LOGOUT

Order Completed

Thank you for your order.

This Agreement between University of Cape Town -- Stefan Barth ("You") and Elsevier ("Elsevier") consists of your license details and the terms and conditions provided by Elsevier and Copyright Clearance Center.

Your confirmation email will contain your order number for future reference.

Printable details.

License Number	4133651242280
License date	Jun 21, 2017
Licensed Content Publisher	Elsevier
Licensed Content Publication	Cancer Letters
Licensed Content Title	A CSPG4-specific immunotoxin kills rhabdomyosarcoma cells and binds to primary tumor tissues
Licensed Content Author	Hannes Brehm,Judith Niesen,Radoslav Mladenov,Christoph Stein,Alessa Pardo,Georg Fey,Wijnand Helfrich,Rainer Fischer,Stefan Gattenlöhner,Stefan Barth
Licensed Content Date	Oct 1, 2014
Licensed Content Volume	352
Licensed Content Issue	2
Licensed Content Pages	8
Type of Use	reuse in a journal/magazine
Requestor type	author of new work
Intended publisher of new work	MDPI AG
Portion	figures/tables/illustrations
Number of figures/tables/illustrations	1
Format	electronic
Are you the author of this Elsevier article?	Yes
Will you be translating?	No
Order reference number	
Original figure numbers	Figure 2
Title of the article	CSPG4: A Target for Selective Delivery of Human Cytolytic Fusion Proteins and TRAIL
Publication new article is in	Biomedicines
Publisher of the new article	MDPI AG
Author of new article	Sandra Jordaan
Expected publication date	Jul 2017
Estimated size of new article (number of pages)	16
Elsevier VAT number	GB 494 6272 12

Requestor Location	University of Cape Town<br>Rm N3.13, Wernher-Beit North<br>UCT Faculty of Health Sciences<br>Anzio Road, Observatory<br>Cape Town, Western Cape 7925<br>South Africa<br>Attn: University of Cape Town
Publisher Tax ID	ZA 4110266048
Total	0.00 USD

ORDER MORE CLOSE WINDOW

Copyright © 2017 Copyright Clearance Center, Inc. All Rights Reserved. Privacy statement. Terms and Conditions.
Comments? We would like to hear from you. E-mail us at customercare@copyright.com
